# Reviewer acknowledgement

**DOI:** 10.1186/1744-8069-9-10

**Published:** 2013-03-22

**Authors:** Jianguo Gu, Min Zhuo

**Affiliations:** 1 Department of Anesthesiology and the Graduate Program in Neuroscience, The University of Cincinnati College of Medicine, PO Box 670531, 231 Albert Sabin Way, Cincinnati, OH, 45267, USA; 2 Department of Physiology, Faculty of Medicine, University of Toronto, 1 King's College Circle; University of Toronto Center for the study of Pain, Toronto, Ontario, M5S 1A8, Canada

## Abstract

**Contributing reviewers:**

The editors of Molecular Pain would like to thank all the reviewers who have contributed to the journal in Volume 8 (2012).

## 

Andrew Ahn

United States of America

Armen Akopian

United States of America

Hakan Aldskogius

Sweden

David Andersson

United Kingdom

A. Vania Apkarian

United States of America

Qassim Aziz

United Kingdom

Alexandru Babes

Romania

Mark Baccei

United States of America

Mark Baker

United Kingdom

Inna Belfer

United States of America

Carlos Belmonte

Spain

Kelly Berg

United States of America

Andreas Beutler

United States of America

Knut Biber

Germany

Fiona Boissonade

United Kingdom

David Borsook

United States of America

Yves Boucher

France

Tim Brennan

United States of America

David Brown

United Kingdom

Peter Cabot

Australia

Yu-Qing Cao

United States of America

Susan Carlton

United States of America

Michael Caterina

United States of America

Guido Cavaletti

Italy

Myeounghoon Cha

United States of America

Hee-Jung Cho

Korea South

MacDonald Christie

Australia

Man-Kyo Chung

United States of America

Mark S Cooper

United States of America

Barbara Costa

Italy

Theodore Cummins

United States of America

Yi Dai

Japan

Alexander Davies

Korea South

Brian Davis

United States of America

Lucy Donaldson

United Kingdom

Xinzhong Dong

United States of America

Jonathan Dostrovsky

Canada

Patrick Dougherty

United States of America

Liam Drew

United States of America

Peter Drummond

Australia

Paul Durham

United States of America

Greg Dussor

United States of America

Lars Edvinsson

Sweden

Nobuaki Egashira

Japan

Ni Fan

United States of America

Jill Fehrenbacher

United States of America

Roger Fillingim

United States of America

Sue Fleetwood-Walker

United Kingdom

Clemens Forster

Germany

Kaj Fried

Sweden

Hidemasa Furue

Japan

Louis Gendron

Canada

Sandrine Geranton

United Kingdom

Urs Gerber

Switzerland

Michael Gold

United States of America

Peter Goldstein

United States of America

Elena Gorodetsky

United States of America

Cyril Goudet

France

Peter Grace

Australia

Yanping Gu

United States of America

Kathryn Hall

United States of America

Liang Han

United States of America

Menachem Hanani

Israel

Kenneth Hargreaves

United States of America

Afton Hassett

United States of America

Quinn Hogan

United States of America

Tomas Hokfelt

Sweden

Fiona Holmes

United Kingdom

Yuuichi Hori

Japan

Hongzhen Hu

United States of America

Li-Yen Huang

United States of America

Mark Hutchinson

Australia

Sun Wook Hwang

Korea South

Soichiro Ide

Japan

Hiroshi Ikeda

Japan

Julia Inglis

Australia

Seiji Ito

Japan

Akitoshi Ito

Japan

Koichi Iwata

Japan

Il-Sung Jang

Korea South

Michael Jarvis

United States of America

Ru-Rong Ji

United States of America

Elizabeth Joseph

United States of America

James Kang

United States of America

Fusao Kato

Japan

Bradley Kerr

Canada

Norikazu Kiguchi

Japan

Shiroh Kishioka

Japan

Kohei Koga

Canada

Toru Kono

Japan

Michaela Kress

Austria

Takashi Kurihara

Japan

Malin Lagerstrom

Sweden

C. Justin Lee

Korea South

Jon Levine

United States of America

Yun-Qing Li

China

Jie Li

United States of America

Ellen Lumpkin

United States of America

ZhengLiang Ma

China

Qiufu Ma

United States of America

Valerio Magnaghi

Italy

Patrick Mantyh

United States of America

Jianren Mao

United States of America

Marco Martina

United States of America

Koichi Masuda

United States of America

Satoshi Matsuoka

Japan

David McKemy

United States of America

Feng Mei

China

Karl Messlinger

Germany

Gregory Michael

United Kingdom

Vjekoslav Miletic

United States of America

Erin Milligan

United States of America

Masabumi Minami

Japan

Jeffrey Mogil

Canada

Yasuo Mori

Japan

Takayuki Nakagawa

Japan

Hiroyuki Nakai

United States of America

Terumasa Nakatsuka

Japan

Ian Napier

Australia

Joseph Neale

United States of America

Timothy Ness

United States of America

Volker Neugebauer

United States of America

Mami Noda

Japan

Koichi Noguchi

Japan

Hideaki Obata

Japan

Uhtaek Oh

Korea South

Seog Bae Oh

Korea South

Masahiro Ohsawa

Japan

Enza Palazzo

Italy

Zhizhong Pan

United States of America

Michele Papa

Italy

Daniela Parolaro

Italy

Ardem Patapoutian

United States of America

Shane Perrine

United States of America

Jeffrey Petruska

United States of America

Olga Pol

Spain

Louis Premkumar

United States of America

Theodore Price

United States of America

Li-Ya Qiao

United States of America

Ke Ren

United States of America

Frank L. Rice

United States of America

Meredith Robbins

United States of America

Edgar Alfonso Romero-Sandoval

United States of America

Shivani Ruparel

United States of America

Anthony Rush

Sweden

Paola Sacerdote

Italy

Michael Salter

Canada

Jana Sawynok

Canada

Martin Schmelz

Germany

Philippe Seguela

Canada

Kenji Seo

Japan

Barry Sessle

Canada

Hari Shanker

Sweden

Bing Shen

China

Shannon Shields

United States of America

Bai Chuang Shyu

Taiwan

Robert Sikes

United States of America

Donald Simone

United States of America

Claudia Sommer

Germany

Martin Stebbing

Australia

Emanuel Strehler

United States of America

Sarah Sweitzer

United States of America

Lauren Ta

United States of America

Yuan-Xiang Tao

United States of America

Isaura Tavares

Portugal

Andrew Todd

United Kingdom

Makoto Tominaga

Japan

Carole Torsney

United Kingdom

Makoto Tsuda

Japan

Hiroshi Ueda

Japan

Magda Ulrich

Netherlands

Mark Urban

United States of America

Yuriy Usachev

United States of America

Todd Vanderah

United States of America

Pedro Vera

United States of America

You Wan

China

Christopher Watson

United States of America

Feng Wei

United States of America

Fletcher White

United States of America

Beth Winkelstein

United States of America

John Wood

United Kingdom

Long-Jun Wu

United States of America

David Wynick

United Kingdom

Tian-Le Xu

China

Guang-Yin Xu

China

Ipek Yalcin-Christmann

France

Jay Yang

United States of America

Robert Yezierski

United States of America

Myung ha Yoon

Korea South

Hanns Ulrich Zeilhofer

Switzerland

Xu Zhang

China

Jun-Ming Zhang

United States of America

Hailin Zhang

China

